# *SMARCA4*突变非小细胞肺癌研究进展

**DOI:** 10.3779/j.issn.1009-3419.2024.102.32

**Published:** 2024-09-20

**Authors:** Lishan PENG, Wenzhao ZHONG

**Affiliations:** 510080 广州，广东省肺癌研究所，南方医科大学广东省人民医院（广东省医学科学院）; Guangdong Lung Cancer Institute, Guangdong Provincial People’s Hospital (Guangdong Academy of Medical Sciences), Southern Medical University, Guangzhou 510080, China

**Keywords:** 肺肿瘤, SMARCA4, SWI/SNF, 免疫治疗, 合成致死, Lung neoplasms, SMARCA4, SWI/SNF, Immunotherapy, Synthetic lethality

## Abstract

非小细胞肺癌（non-small cell lung cancer, NSCLC）是全球发生率和死亡率最高的癌症之一。尽管精准治疗模式下靶向药物及免疫药物的应用改善了患者的预后，但仍有患者无法从中获益，因此探索耐药原因及机制具有重要意义。生存分析发现SMARCA4突变NSCLC患者预后不佳。SMARCA4是SWI/SNF染色体重塑复合物中具有ATP酶活性的核心亚基，SWI/SNF复合体通过改变染色质可及性调控基因转录，影响分化、细胞周期调控等重要细胞活动，被认为具有抑癌作用。本文将综合阐述SMARCA4突变在肿瘤发生发展过程中的作用以及其在NSCLC中的临床病理特点、治疗预后及潜在治疗方案。

## Abstract (en)

Non-small cell lung cancer (NSCLC) is one of the most prevalent and deadliest cancers worldwide. While the use of targeted therapies and immunotherapies in precision medicine has improved outcomes for some patients, a significant portion of individuals still fail to benefit, emphasizing the need to investigate the underlying mechanisms of resistance. Survival analyses have shown that NSCLC patients with SMARCA4 mutations often have poor prognoses. SMARCA4, the core ATPase subunit of the SWI/SNF chromatin remodeling complex, plays a critical role in regulating gene transcription by modifying chromatin accessibility. This influences essential cellular processes such as differentiation and cell cycle regulation, and SMARCA4 is widely regarded as a tumor suppressor. This review will explore the role of SMARCA4 mutations in tumor progression, its clinicopathological features in NSCLC, its impact on treatment outcomes, and potential therapeutic strategies.

肺癌具有高发病率和死亡率，2022年全球共有250万新发肺癌患者，其中80%为非小细胞肺癌（non-small cell lung cancer, NSCLC），并有180万人因肺癌死亡^[1]^。近年来，驱动基因研究的深入以及靶向和免疫治疗等药物的使用，推动了基于分期、病理、基因突变检测的精准治疗模式形成，改善了患者的预后，但仍有患者面临早期复发和疾病进展。因此伴随基因在疾病发展及治疗中的作用受到日益关注，准确了解不同基因突变在肺癌中的特点及致癌机制对于精准治疗具有重要意义。而SMARCA4就是其中一个重要的伴随基因，SMARCA4基因作为SWI/SNF染色体重塑复合物的核心催化亚基，其突变失活会改变染色质可及性并影响基因的转录调控，形成具有侵袭性的表型，并且与不良预后相关^[2]^。本文综述了SMARCA4突变的机制以及其在NSCLC中的特点、治疗及未来展望。

## 1 SMARCA4基因与SWI/SNF复合物

SMARCA4基因位于19号染色体，编码具有7个结构域的BRG1蛋白，是组成SWI/SNF染色体重塑复合物的核心亚基^[3]^。染色质重塑在基因表达调控中起到重要作用。SWI/SNF复合物识别特定染色质区域后通过ATP酶水解获得能量滑动或移除核小体，展开染色质，招募转录因子并作用于启动子^[4]^和增强子^[5]^，促进转录启动，进而在细胞周期调节^[6]^、细胞分化^[7]^等生物过程中发挥作用，并且被认为是抑癌因子。SWI/SNF复合物还能促进DNA同源重组修复^[8]^，参与DNA损伤位点的核苷酸切除修复^[9]^。SWI/SNF复合物这一识别-调节-驱动的染色质重塑过程需要多个亚基协同参与，在哺乳动物中不同亚基组装形成多种不同亚家族，而BRG1是其中核心亚基，不仅编码驱动模块的ATP酶，SMARCA4内结构域还与其他亚基一起参与识别及调节模块功能^[3]^。因此，当SMARCA4基因发生突变时，可以通过影响ATP酶活性或染色质识别结合功能，进而影响转录及DNA损伤修复^[3]^，并与肿瘤的发生发展相关。在转录调节方面，研究^[10]^证明SMARCA4缺失直接导致所有三类SWI/SNF复合物在调节区域结合和打开染色质的能力缺陷，导致肺谱系转录因子结合的染色质可及性降低。另一方面，SWI/SNF复合物参与组蛋白修饰，与转录抑制因子多梳蛋白抑制复合物（polycomb repressive complexes, PRC）相互拮抗，当SMARCA4突变后导致BRG1无法与PRC结合并将其移除，使得其在启动子等区域大量沉积，使得增强子可及性的下降^[11]^，PRC异常激活已被证实会刺激肿瘤细胞增殖及侵袭^[12]^。在转录上可以观察到由于调控异常引发的变化，突变缺失导致与增殖、血管生成相关基因表达上调，如癌基因MYC^[11]^，而分化、黏附、凋亡等相关的基因表达下调^[13^，如抑癌基因P53^[14]^和Rb^[15]^。SMARCA4缺失还会影响DNA损伤修复，一方面缺失导致复制叉缺陷，DNA无法正确完成复制进而使得DNA损伤增加^[16]^，另一方面缺失导致的染色质重塑障碍影响损伤位点的可及性，阻碍损伤修复过程^[17]^，两者共同作用导致基因组不稳定性增强，推动肿瘤的发展。突变导致转录和DNA损伤修复障碍共同作用于机体，在NSCLC患者^[2]^和动物模型^[10]^可以观察到去分化、高侵袭性、高转移、高肿瘤突变负荷（tumor mutational burden, TMB）的特点，尤其是在SMARCA4缺失组中，而突变如何导致耐药还需要更多的探索。

## 2 SMARCA4突变NSCLC的特征

SMARCA4突变NSCLC在临床、病理等领域与野生型相比都具有不同特点，具有侵袭性及更高比例的去分化状态，并与不良预后相关，而其中胸部SMARCA4缺失的未分化肿瘤（thoracic SMARCA4-deficient undifferentiated tumor, SMARCA4-UT）在2021年被世界卫生组织（World Health Organization, WHO）列为一类特殊表型的上皮来源NSCLC^[18]^，下文将详细描述其特征。

### 2.1 SMARCA4突变NSCLC临床病理特征

SMARCA4突变可以通过高通量测序进行检测，5%-10%的NSCLC患者携带SMARCA4突变^[19⇓-21]^，超过80%患者具有吸烟史，中位年龄约为63岁，突变使得肿瘤更具侵袭性，84.2%患者在确诊时已经出现远处转移，比野生型具有更高的晚期比例^[21]^，并以骨转移、肝转移常见^[22]^。

SMARCA4突变另一个特点是去分化状态在BRG1缺失患者中更显著，然而SMARCA4突变和蛋白缺失并不一一对应，40%-80%的SMARCA4突变NSCLC会出现BRG1蛋白的缺失^[20,23]^，由于表观遗传改变以及技术局限导致的染色体异常如重排、错位等无法识别，35%的BRG1缺失患者中没有检测到SMARCA4突变^[23]^。在携带SMARCA4突变且免疫组化明确蛋白表达缺失的患者与突变型患者具有相似的临床特征，其病理类型虽然同样以腺癌常见^[20,21]^，但具有更高比例的非小细胞癌未另行说明（not otherwise specified, NOS）类型（14%^[20]^ vs 3.9%^[21]^）。暂无文献报道SMARCA4突变NSCLC的病理学特征，而BRG1缺失型在镜下形态和免疫组化染色上与野生型腺癌具有较大差异，不同于腺癌具有明显的腺体或腺泡样结构，缺失型腺癌表现为少量未成熟小腔和散在的杯状细胞，细胞具有多形性，68%可见横纹肌样细胞^[24,25]^。甲状腺转录因子-1（thyroid transcription factor 1, TTF-1）、新天冬氨酸蛋白酶A（new aspartic proteinase A, Naspin A）腺癌标志物只有在少量具有腺癌分化的区域或BRG1表达的区域呈阳性，多数为阴性，但上皮标志物细胞角蛋白7（cytokeratin 7, CK7）常为阳性^[26]^。除此之外，缺失型患者免疫组化中可见检测出肝细胞标志物HepPar-1强阳性，虽然暂无研究说明HepPar-1与突变之间的内在联系，HepPar-1可以作为与野生型腺癌区分的标志之一^[24,25]^。

### 2.2 SMARCA4突变NSCLC分子学特征

SMARCA4最常见的突变类型为错义突变，约占总比例的50%，是Schoenfeld定义的II类突变的主体^[19]^，虽然较少导致表达缺失，但是研究表明错义突变会导致SWI/SNF复合体重塑功能异常，降低染色质可及性，包括增强子和超级增强子可及性，其中以G1192C、G1232S、G1162C导致关闭染色质位点最多^[2]^，另一方面突变也会影响SWI/SNF的识别功能，如R1372A突变影响精氨酸锚点与组蛋白识别^[3]^。无义突变和移码突变是仅次于错义突变的形式，分别占比22%和12%，构成I类突变的主要部分，与蛋白缺失相关^[19]^。突变类型会影响生存，这将在后文阐述。

除突变类型，共突变也是影响因素，最常见的共突变是TP53突变，56%患者携带此共突变，也位于19号染色体的KEAP1、STK11突变概率为41%和39%，驱动基因突变中最常见的是KRAS，近1/3的患者携带共突变，而表皮生长因子受体（epidermal growth factor receptor, EGFR）只有14%^[19]^，被认为与SMARCA4突变互斥^[2]^，后文将进一步描述共突变如何影响生存。

### 2.3 SMARCA4突变NSCLC免疫微环境特征

由于免疫治疗的应用，与疗效相关的预测因子受到关注。与野生型相比，SMARCA4突变时程序性细胞死亡配体1（programmed cell death ligand 1, PD-L1）多为低表达或阴性^[19,27]^，而呈现高TMB（P<0.001）^[19]^，当携带多个SWI/SNF突变时TMB可超过20个/MB^[21]^。虽然在肺腺癌中高TMB经常与更好的免疫治疗反应率相关，但这一现象背后机制是肺腺癌中TMB与CD8细胞浸润呈正相关^[28]^，而SMARCA4突变使得CD8^-^ T细胞浸润减少^[29]^，当携带KRAS共突变时，多重免疫荧光检测可以观察到程序性细胞死亡受体1（programmed cell death 1, PD-1）^+^细胞以及PD-1^+^CD8^+^细胞减少^[21]^，FOXP3^+^细胞和中性粒细胞浸润增加^[30]^。在Chen等^[31]^构建的TMEsig-score免疫微环境评分系统中，SMARCA4突变在低分亚组中聚集（13% vs 4%），低评分的TME-C3组被认为是以肿瘤细胞增殖、糖酵解和免疫抑制为表现的免疫沙漠表型。以上提示SMARCA4突变患者可能处于免疫抑制的微环境中。

### 2.4 SMARCA4-UT的特征

SMARCA4-UT较SMARCA4缺失NSCLC更有侵袭性及更差的分化状态。患者患病中位年龄为48岁，只有约10%患者不具有吸烟史^[18]^，影像学可见巨大肿瘤平均直径为9.2 cm（范围：2.2-18.3 cm），多累及肺与纵隔，91%的患者就诊时已为IV期^[32]^，由于肿物巨大，患者可出现呼吸困难、疼痛、上腔静脉综合征等症状。未分化状态在镜下表现为弥漫性核仁突出的单一形态圆形或者上皮样细胞，常可见横纹肌样细胞，并且几乎找不到上皮分化区域^[18]^。免疫组化上除BRG1表达缺失和腺体表达TTF-1、Claudin-4、pan-CK的缺失外，SMARCA4-UT患者还同时可见BRM蛋白缺失，血管内皮标志物CD34呈阳性。BRM蛋白是SWI/SNF复合物中同样承担ATP酶功能的亚基，与BRG1功能互斥，BRG1和BRM缺失会导致SWI/SNF复合物的完全丧失，有假说^[32]^提出SMARCA4缺失NSCLC可能在经历二次突变打击后使得BRM蛋白缺失转变为分化更差的SMARCA4-UT。

## 3 SMARCA4突变NSCLC的治疗和预后

### 3.1 可手术SMARCA4突变NSCLC

SMARCA4突变也是可手术NSCLC患者的术后不良预后的风险因素^[22]^，在纳入42例I、II期术后NSCLC患者的研究^[33]^中，突变组无复发生存时间（recurrence free survival, RFS）显著下降（突变组 vs 野生组：4.5 vs 28.5个月，P=0.0007）。JBR.10试验的事后分析显示，无论患者分期和组织学情况，相比术后观察，BRG1低表达患者可以从术后辅助顺铂中获益^[34]^。现暂无数据报道SMARCA4突变对新辅助免疫或辅助免疫治疗的影响，而突变在根治性治疗后仍早期复发的机制还需要进一步研究。

### 3.2 晚期SMARCA4突变NSCLC

与野生型患者比较，晚期SMARCA4突变患者总体对于治疗不敏感^[20]^，突变型患者总生存期（overall survival, OS）为15.6个月，显著低于野生型的25.6个月（P<0.01）^[21]^。进一步对突变患者做亚组分析，在突变类型上I类突变患者预后最差^[19]^（HR=1.59, 95%CI: 1.25-2.04），尤其是纯合截短突变^[2]^（HR=1.85, 95%CI: 1.47-2.34）。共突变中合并KRAS^[35]^或STK11或KEAP1^[19]^时OS更差。

与其他治疗相比，免疫治疗可以延长突变患者OS^[19,36]^，BRG1缺失NSCLC患者在一线免疫联合化疗后无进展生存期（progression-free survival, PFS）为8.2个月，显著优于单纯化疗的3.5个月^[36]^。有意思的是，早期手术后患者可以从辅助化疗中获益，但晚期患者对单纯化疗显示出耐药性，尽管都存在SMARCA4突变，但不同分期的异质性可能使得药物在体内作用机制改变。虽然免疫治疗取得更好疗效，但SMARCA4突变与一线免疫联合化疗的不良预后相关，美国纽约纪念Sloan-Kettering癌症中心（Memorial Sloan-Kettering Cancer Center, MSKCC）联合达纳-法伯癌症研究所（Dana-Farber Cancer Institute, DFCI）的707例非鳞癌晚期NSCLC数据显示SMARCA4突变亚组对一线免疫联合化疗不敏感[突变型 vs 野生型：客观缓解率（objective response rate, ORR）21.9% vs 39.1%，P<0.001；中位PFS 2.7 vs 6.1个月，HR=1.62（95%CI: 1.30-2.01），P<0.001；中位OS 8.1 vs 15.0个月，HR=1.70（95%CI: 1.33-2.17），P<0.001]^[37]^。当不限制治疗线数，统计至少接受1个周期免疫单药治疗患者时，突变型患者与野生型患者PFS、OS没有明显差异^[21]^（突变型 vs 野生型：PFS 2.1 vs 3.1个月，P=0.62；OS 11.0 vs 12.4个月，P=0.25）。虽然没有文献单独统计后线免疫治疗疗效，有个案报道中多线治疗仍可以从免疫治疗中获益，如1例IVB期患者二线免疫治疗后维持，OS超过4年^[38]^。一线治疗和多线治疗的差异是否与SMARCA4突变的原发性和获得性相关，需要进一步研究。这也说明SMARCA4突变患者中仍存在很大的异质性，只有部分患者能从免疫治疗中获益。免疫治疗亚组分析中，不限线数时I类和II类突变没有显著影响PFS或OS^[19]^，而纯合截短突变使得患者在免疫治疗后OS较野生型显著下降^[2]^。另一方面以共突变分组时，在小样本的数据中只携带SMARCA4突变患者较野生型PFS延长^[39]^，然而这需要大样本的进一步证实。合并KRAS突变^[21,40]^、KEAP1^[41]^、STK11^[21]^已被证实与更差的预后相关，其中KRAS合并SMARCA4可见CD8 T细胞浸润减少^[21]^，而KEAP1、STK11是肿瘤免疫负性突变^[42]^，形成了抑制性肿瘤微环境，更容易出现免疫原发耐药。有2项临床试验（NCT05286801、NCT04416568）探索不同免疫检查点药物联合是否能够改善SMARCA4缺失肿瘤患者预后。

SMARCA4突变靶向治疗数据较少，但都与不良预后相关，在使用间变性淋巴瘤激酶-酪氨酸激酶抑制剂（anaplastic lymphoma kinase-tyrosine kinase inhibitor, ALK-TKI）的脑转移患者中，携带SMARCA4共突变与更差的预后相关^[43]^，而在KRAS G12Ci单药治疗中SMARCA4突变是影响治疗的独立预后因素^[44]^，EGFR驱动基因突变合并BRG1缺失患者应用EGFR-TKI也易发生早期耐药和转移^[20]^。而有研究^[45]^表明SWI/SNF调控奥希替尼耐药相关基因，敲除BRG1后，部分耐药模型对于奥希替尼重新敏感，SMARCA4在靶向治疗中的作用仍需要更多探索。

### 3.3 SMARCA4-UT患者预后

SMARCA4-UT患者对放化疗或单纯手术反应差，中位生存时间仅为7个月^[18]^。目前报道的数据中UT患者对免疫治疗敏感，在可切除患者中接受新辅助免疫联合化疗的患者预后优于单纯手术或者辅助化疗，个案报道中1例接受新辅助帕博利珠单抗+白蛋白紫杉醇+卡铂，手术病理达到病理完全缓解，术后10个月随访未出现复发转移^[46]^，而1例直接行根治性手术IIB期患者在术后2个月出现脑转移，并在确诊4个月后死亡^[47]^，另有报道即使接受根治性术且术后接受辅助化疗或放疗，患者仍早期复发转移，中位无病生存期（disease-free survival, DFS）仅为4.8个月。晚期患者免疫治疗依然是敏感的，一项回顾性研究^[46]^分析13例接受一线治疗的UT患者，7例免疫联合治疗患者中位PFS较6例化疗患者显著提升（26.8 vs 2.73个月，P=0.044），后线接受免疫治疗效果依然优于化疗^[46]^，另一项回顾性研究^[48]^探讨5例一线接受免疫单药/免疫联合患者无论PD-L1表达情况，患者影像学评估都达到了部分缓解，中位PFS未达到。但是样本数量少，还需更多的数据来说明免疫治疗的疗效。

## 4 SMARCA4突变潜在治疗方案

### 4.1 合成致死

两个非致死基因表达同时被抑制导致的细胞死亡被称为合成致死，通过这一机制可以杀伤具有突变的肿瘤细胞而减少对于正常细胞的影响，实现精准靶向。因此突变对应合适的合成致死靶点成为关键。现已发现多个SMARCA4潜在合成致死靶点。

#### 4.1.1 SMARCA2靶点

首先发现的靶点是SMARCA2。SMARCA2是SWI/SNF复合物中与SMARCA4互补的ATPase酶，当SMARCA4功能丧失时，SMARCA2对于维持细胞形态和细胞周期具有重要作用，当继续沉默SMARCA2，细胞周期停滞并且诱导细胞衰老死亡，而在无突变的细胞中生长增殖不受影响^[2]^。因此，SMARCA2被认为是可行的靶点，针对BRM中溴结构阈的抑制剂PFI-3在体外实验中未能展现SMARCA2表达抑制以及抗增殖作用^[49]^，以蛋白水解靶向嵌合体（proteolysis targeting chimera, PROTAC）技术开发的药物对BRG1缺失肿瘤在临床前的体内外显示强大的抗瘤效果^[50]^。另一种降解剂PRT3789单药或联合多西他赛在SMARCA4突变实体瘤患者中开展I期临床试验（NCT05639751）。如果实现BRM表达完全抑制，是否会有突变导致患者向更具侵袭性的UT转换，需要更多数据说明药物安全性。

#### 4.1.2 共济失调毛细血管扩张和Rad3相关激酶（ataxia telangiectasia and Rad3-related, ATR）靶点

SMARCA4和ATR共同参与DNA损伤修复，并被认为具有合成致死的关系。BRG1蛋白缺失后，损伤DNA增加及其与复制蛋白A高效结合使得ATR通路激活而促进DNA修复以维护染色质稳定性，当ATR被抑制时，复制叉功能进一步受阻，基因不稳定性增加导致DNA断裂细胞死亡，体内外实验^[16]^也证实SMARCA4缺失型对ATR抑制剂敏感。虽然暂无专门针对SMARCA4突变或缺失型NSCLC患者的临床试验，但是有多款ATR抑制剂在NSCLC患者中开展试验，Ceralasertib的II期伞式研究HUDSON证实了其联合免疫治疗在免疫联合化疗失败的患者中有良好的疗效[ORR 13.9%（11/79），OS 17.4个月（95%CI: 14.1-20.3）]^[51]^。并开展III期临床试验LATIFY以更进一步比较联合治疗和二线治疗多西他赛疗效（NCT05450692），期待亚组分析结合下一代测序（next-generation sequencing, NGS）对于疗效评估的判断。

#### 4.1.3 周期蛋白依赖性激酶4/6（cyclin-dependent kinase 4/6, CDK4/6）靶点

在SMARCA4参与的细胞周期调节中也发现了合成致死靶点CDK4/6。细胞周期从G_1_期转向S期需要细胞周期蛋白D1（cyclin D1, CCND1）和CDK4/6结合。SMARCA4的缺失导致通过限制CCND1染色质的可及性和表达，继续抑制CDK4/6导致的细胞周期阻滞，细胞没有进入DNA修复而是凋亡^[52]^。虽然CDK4/6抑制剂已经被美国食品药品监督管理局（Food and Drug Adminstration, FDA）批准应用于晚期乳腺癌的治疗，但单药和联合治疗在后线的NSCLC均未达到预期疗效^[53]^，联合免疫治疗展现较强的药物毒性^[54]^，CDK4表达增高不能作为生物标志物（NCT02664935），SMARCA4能否作为标志物需要临床试验数据的支撑。

#### 4.1.4 其他靶点

文库筛选发现参与有丝分裂的Aurora激酶A（aurora kinase A, AURKA）也是SMARCA4合成致死靶点，在临床前试验中证实了SMARCA4缺失肺癌对于AURKA抑制剂敏感^[55]^。目前在NSCLC中仅在EGFR突变患者中联合奥希替尼开展I期临床试验。除此之外SMARCA4/2缺失抑制葡萄糖转运蛋白GLUT1的表达，导致葡萄糖摄取和糖酵解减少，对氧化磷酸化（oxidative phosphorylation, OXPHOS）途径获能的依赖性升高，SMARCA4/2缺陷的癌细胞对靶向OXPHOS抑制剂敏感^[56]^。组蛋白去甲基化酶抑制剂GSK-J4对SMARCA4缺失细胞具有强烈的抗肿瘤效果^[57]^，虽然都暂未有临床试验，但也可以是未来探索的方向。

### 4.2 表观遗传拮抗

SWI/SNF复合体参与组蛋白修饰，与转录抑制因子PRC相互拮抗，而组蛋白甲基转移酶（enhancer of zeste homolog 2, EZH2）是PRC2的催化亚基，EZH2在第27位赖氨酸（H3K27甲基化）对组蛋白H3进行三甲基化并诱导基因沉默，通过抑制过度活跃的EZH2功能，调节参与细胞周期调控和终末分化基因的转录，从而抑制肿瘤细胞增殖^[12]^。BRG1功能缺失NSCLC细胞系在EZH2抑制剂应用背景下，增加S期细胞比例、细胞凋亡比例，并增强拓扑异构酶II抑制剂抑制敏感性^[58]^。纳入I期临床试验的3例SMARCA4缺失的实体瘤患者，2例卵巢高钙血症型小细胞癌患者病灶稳定或达到部分缓解，第3例SMARCA4-UT患者疗效不佳^[59]^。另有一项针对儿童INI/SMARCA4蛋白缺失实体肿瘤患者评估联合他泽司他、纳武利尤单抗、伊匹木单抗疗效的I/II期临床试验正在招募中（NCT05407441）。

虽然暂无一项研究专门针对SMARCA4突变/缺失NSCLC患者，但期待这些试验中亚组结果能够推进我们对于突变及缺失的认知以及治疗策略，也期待后续针对SMARCA4突变的临床试验。

## 5 总结

由于染色质可及性的改变，SMARCA4突变NSCLC患者具有特别的的表型并与预后差相关，虽然免疫治疗相较其他治疗方案更有效，但回顾性研究发现SMARCA4突变仍是晚期一线免疫联合化疗复发的独立危险因素，而后线免疫治疗可能不受影响，这需要前瞻性试验和对突变作用机制的深入研究，以识别真正能够从免疫治疗中获益的患者。临床前体内外试验发现了针对SMARCA4突变的新药靶点，有些药物正在NSCLC中进行临床试验，期待这些研究结果能够推进我们对SMARCA4的认知，并推动专门针对SMARCA4突变患者的临床试验。
